# Unmasking small-bowel DLBCL in an elderly patient with simultaneous multiple primary cancers through anemia: a case report and literature review

**DOI:** 10.3389/fonc.2026.1706363

**Published:** 2026-02-19

**Authors:** Binlin Da, Chen Zhao, Yao Yao, Chengting Dai, Nan Zhu, Wei Yan, Juanjuan Zhang, Jun Jiang, Zhiming Wang, Lei Guo

**Affiliations:** 1 Research Institute of General Surgery, Jinling Hospital, Affiliated Hospital of Medical School, Nanjing University, Nanjing, Jiangsu, China; 2 Department of Endoscopy, Jinling Hospital, Affiliated Hospital of Medical School, Nanjing University, Nanjing, Jiangsu, China; 3 Department of Pathology, Jinling Hospital, Affiliated Hospital of Medical School, Nanjing University, Nanjing, Jiangsu, China; 4 Cadre Ward Section One, Jinling Hospital, Affiliated Hospital of Medical School, Nanjing University, Nanjing, Jiangsu, China

**Keywords:** anemia, cardia adenocarcinoma, ileum diffuse large B-cell lymphoma, lung adenocarcinoma, multiple primary cancers

## Abstract

Multiple primary cancers (MPC) refer to the occurrence of two or more histologically distinct tumor types in a single individual, either simultaneously or sequentially. This report presents a rare and instructive case of a 71-year-old male who developed primary small intestinal diffuse large B-cell lymphoma (DLBCL) nine years after curative treatment for synchronous cardia and lung adenocarcinomas. The patient presented with dizziness and fatigue. Initial evaluation at a local hospital revealed moderate anemia (hemoglobin 66 g/L). Esophagogastroduodenoscopy and colonoscopy showed no remarkable findings. Subsequently, capsule endoscopy was performed. The capsule endoscopy demonstrated a segment of abnormal small bowel mucosa, showing marked edema, extensive erosions, and deep ulcers. The patient was then admitted to our center for further diagnosis and treatment. Chest computed tomography (CT) demonstrated postoperative changes from the prior lung and cardia cancers. Contrast-enhanced abdominal CT revealed focal ileal wall thickening and multiple small lymph nodes, suggestive of a neoplastic lesion. Further investigation was recommended. Single-balloon enteroscopy revealed a circumferential neoplastic growth, approximately 10 cm in length, with an ulcerative appearance and a dirty purulent coating in the ileum. Biopsy of the ileal lesion revealed a malignant lymphohematopoietic tumor with ulceration and necrosis, which on immunohistochemistry supported a diagnosis of aggressive B-cell lymphoma. Then the patient completed positron emission tomography - computed tomography (PET-CT) showing primary small-bowel lymphoma, Lugano staging 1E. To alleviate anemia and reduce the tumor burden, a partial resection of the ileum was performed. The segmental ileal resection confirmed DLBCL. Postoperatively, the patient was advised to be transferred to the oncology department for R-CHOP (rituximab, cyclophosphamide, doxorubicin, vincristine, and prednisone) chemoimmunotherapy. This case highlights the diagnostic challenge of evaluating new symptoms in cancer survivors and underscores the critical importance of considering metachronous hematologic malignancies, even with atypical presentations like isolated anemia, to avoid anchoring bias towards prior solid tumors.

## Introduction

Over the past two decades, the frequency of patients presenting with two or more anatomically and temporally distinct primary malignancies—commonly termed multiple primary cancers (MPC)—has been steadily increasing worldwide, currently representing 2–17% of all newly diagnosed tumors (1, 2). The biological basis for MPC is multifactorial, involving a complex interplay among viral infections, cumulative environmental exposures, modifiable lifestyle factors, such as chewing betel nuts and smoking, chronic immune dysregulation, and the carcinogenic sequelae of prior oncological therapies (3–6). The literature on MPC often focuses on combinations of solid tumors, the subsequent development of a hematologic malignancy following dual solid primaries is exceptionally rare and presents a unique diagnostic dilemma. Patients with a history of cancer are at a perpetually elevated risk for new malignancies; however, when non-specific symptoms like anemia arise, clinical reasoning can be inadvertently constrained by “anchoring bias”. This cognitive bias leads to an over-reliance on the pre-existing medical narrative, often attributing new symptoms to the recurrence or late effects of known prior cancers, which can delay the diagnosis of a distinct, new disease entity. Lymphoma is a heterogeneous entity that includes Hodgkin’s lymphoma (HL) and non-Hodgkin’s lymphoma (NHL) (7). Diffuse large B-cell lymphoma (DLBCL) is the most common type of primary small intestinal lymphoma, with about 25% to 30% of patients presenting with localized disease at initial diagnosis (8). DLBCL represents one-third of all NHL cases, with incidence rates varying from 20% to 50% across different countries (9). The risk of developing DLBCL increases monotonically with age, and in all racial categories, the incidence rate is higher in males than in females. Individuals of white race have the highest incidence of DLBCL (10–12).

Herein, we present a case that exemplifies this diagnostic dilemma: a patient with a history of synchronous cardia and lung adenocarcinomas who, nine years later, presented with progressive dizziness and fatigue, ultimately diagnosed with primary small bowel DLBCL.

## Case presentation

### General information

A 71-year-old male patient was admitted to the local hospital due to progressive dizziness, fatigue, and a weight loss of 6 kg over the past two months. Initial evaluation revealed moderate anemia (hemoglobin 66 g/L). Esophagogastroduodenoscopy and colonoscopy showed no remarkable findings, prompting the performance of capsule endoscopy. The capsule transit lasted 14 hours and 44 minutes. From 7 hours and 24 minutes to 12 hours and 57 minutes, the capsule traversed a segment of small bowel characterized by marked mucosal edema, extensive erosions and deep ulcers (**Supplementary Figure 1**). The patient was admitted to our center for further diagnosis and treatment. The patient’s past oncological history was notable for two metachronous primary malignancies. In December 2016 the patient underwent curative-intent total gastrectomy for a poorly differentiated adenocarcinoma of the gastroesophageal junction (pT3N1M0). Histology showed invasion into the muscularis propria with focal extension to the serosa, lymphovascular invasion, and metastasis in 2 of 14 perigastric lymph nodes. At the same time, a wedge resection of the left upper lobe was performed for a 2-cm, moderately differentiated invasive adenocarcinoma classified as lung primary (pT1bN0M0). Regular chemotherapy was administered postoperatively.

### Treatment course

**Figure 1:**
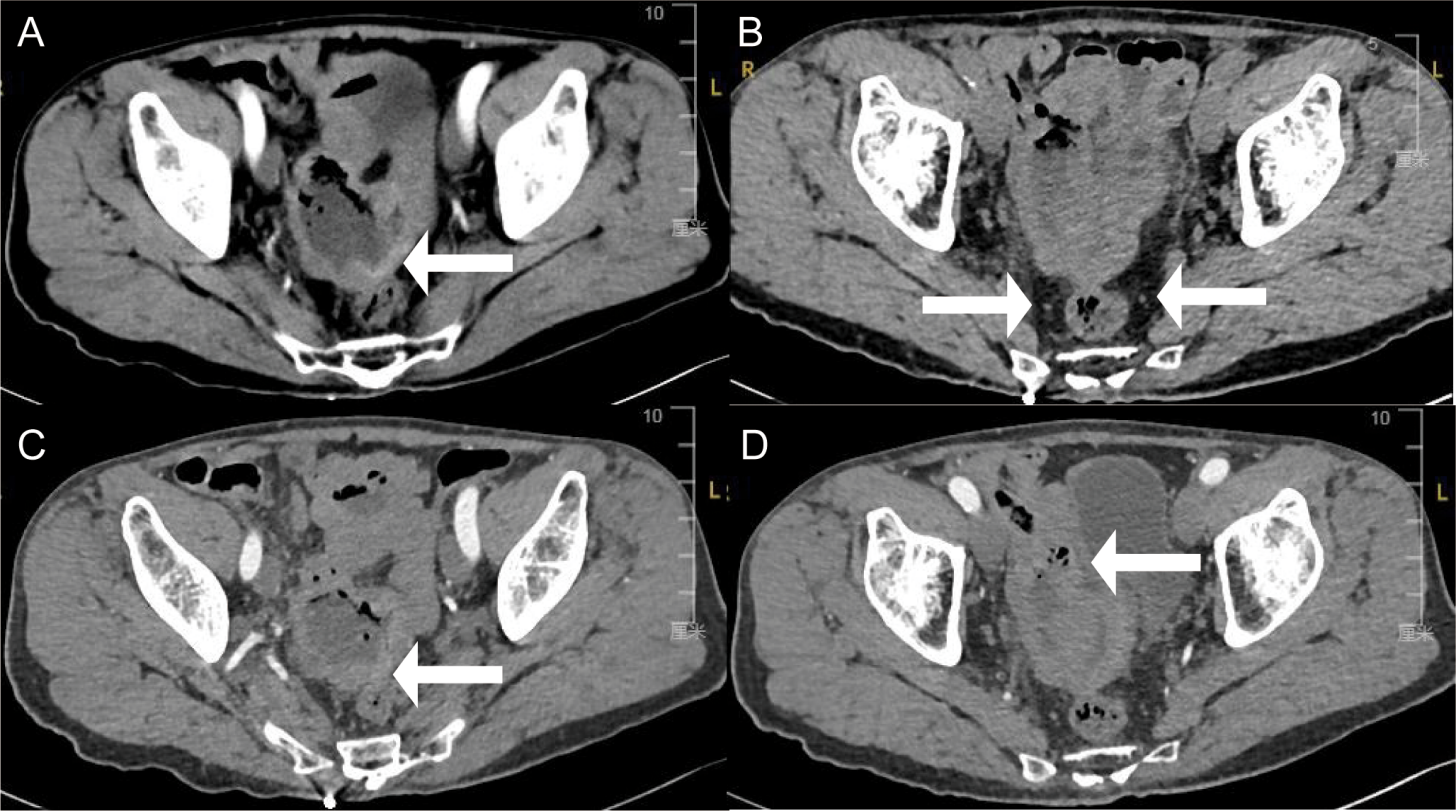
Enhance abdominal computed tomography cross-section images showing **(A)** thickening of part of the ileal wall (white arrows), **(B)** multiple small lymph nodes around the lesion (white arrows), **(C)** indistinct surrounding spaces with the sigmoid colon (white arrows), and **(D)** closely to the right posterior bladder wall (white arrows).

**Figure 2:**
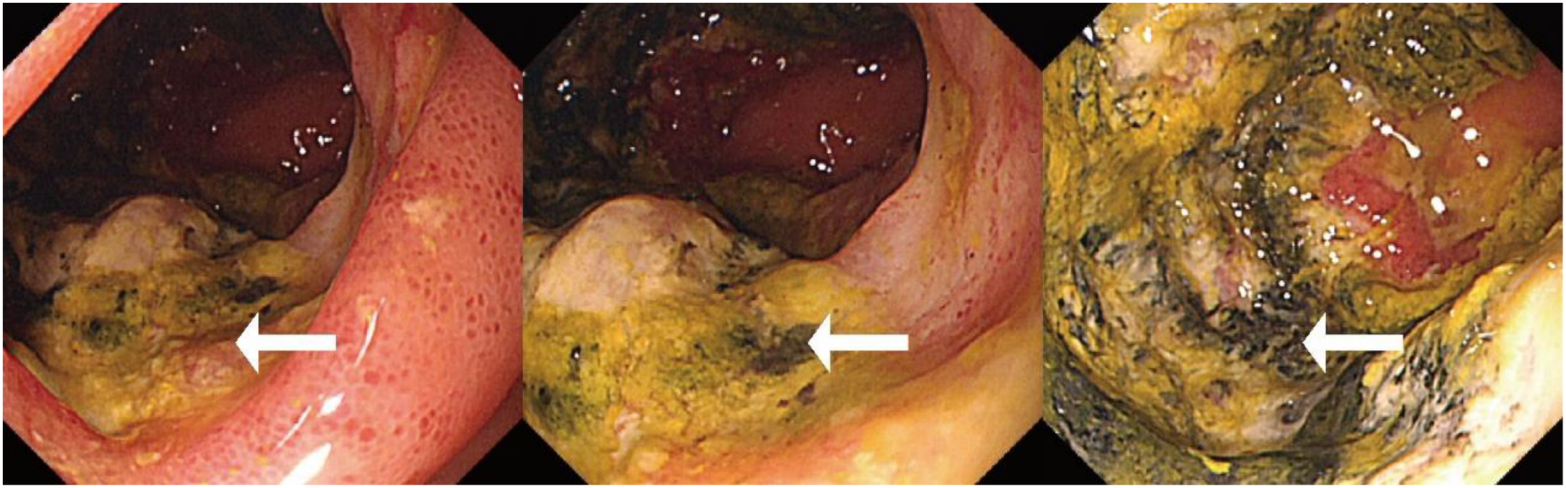
Single-balloon enteroscopy showing a circumferential neoplastic growth, approximately 10 cm in length, with an ulcerative appearance and a dirty purulent coating in the ileum (white arrows).

**Figure 3:**
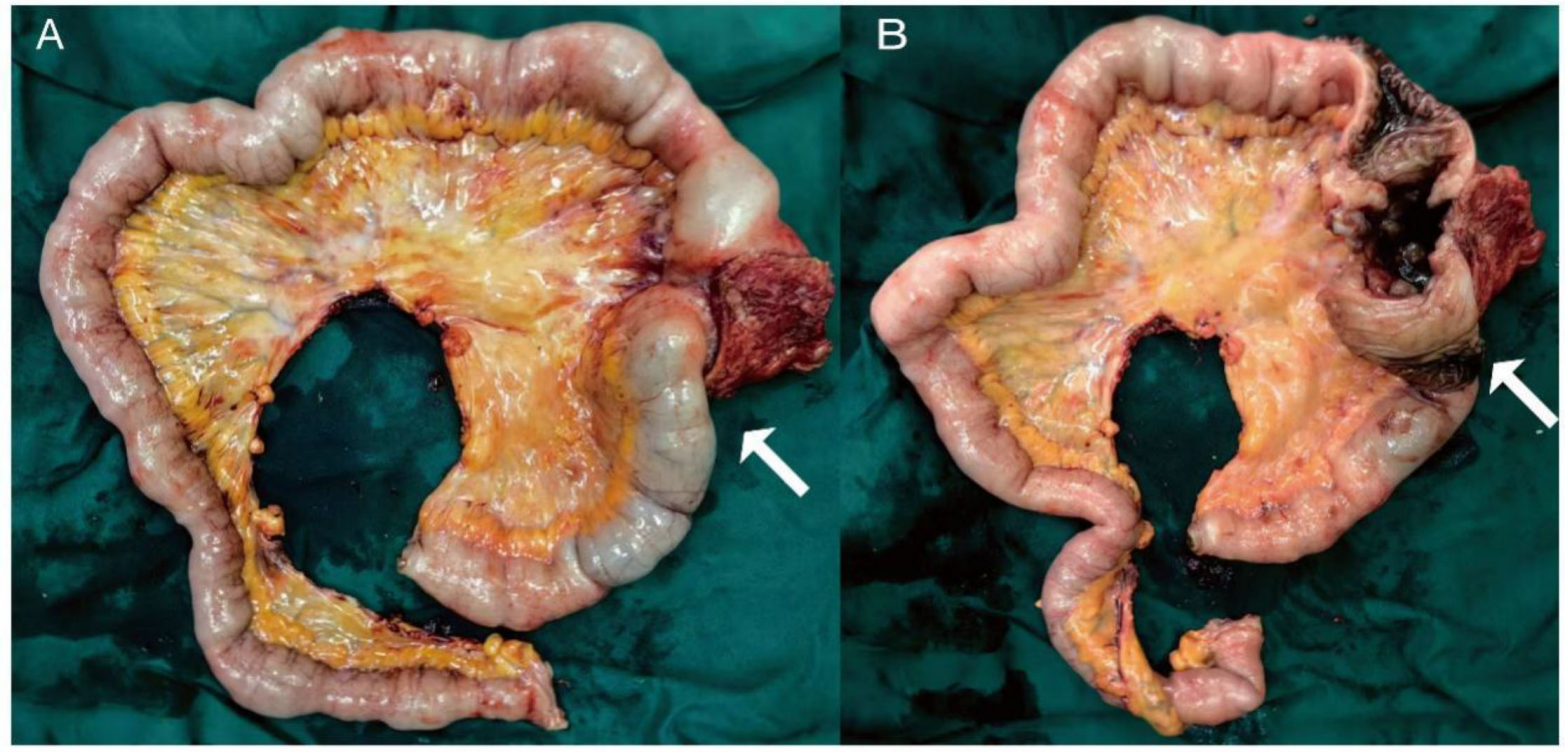
The gross specimen of the surgery **(A)** an 11-cm-long mass observed in the ileum (white arrows), **(B)** appearance of the mass after dissection.

**Figure 4:**
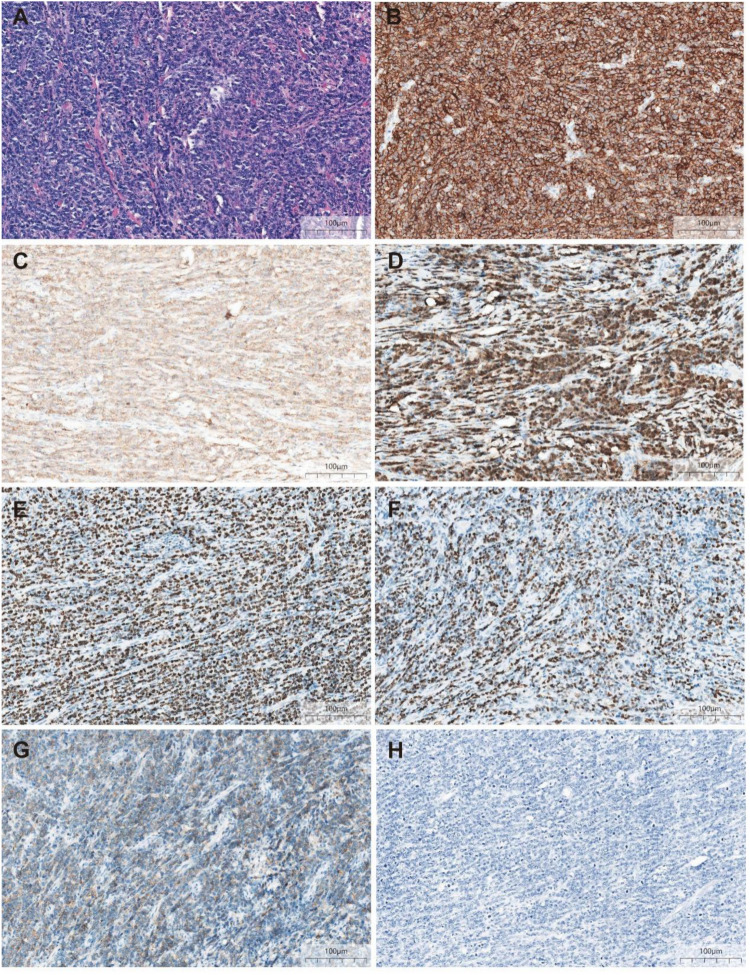
Postoperative pathology **(A)** ileum with a dense and diffuse large lymphocytic infiltrate (HE, ×200) **(B)** Atypical cells diffusely positive for marker CD20 (IHE CD20, ×200) **(C)** Atypical cells diffusely positive for marker CD79a (IHE CD79a, ×200) **(D)** Atypical cells diffusely positive for marker Pax-5 (IHE Pax-5, ×200) **(E)** Ki-67 hot spots at about 80% (+) (IHE Ki-67, ×200) **(F)** Atypical cells partly positive for marker BCL-6 (IHE BCL-6, ×200) **(G)** Atypical cells partly positive for marker CD10 (IHE CD10, ×200) **(H)** EBER *in situ* hybridization (-) (×200).

Initial investigations at our center identified anemia (hemoglobin 89 g/L) and a positive fecal occult blood test. Imaging studies were subsequently performed. Chest computed tomography (CT) revealed postoperative changes of left upper lobe lung cancer and cardia cancer, with scattered solid nodules present in the upper lobes of both lungs and the right middle lobe. Contrast-enhanced abdominal CT further indicated a suspicious ileal lesion, characterized by wall thickening, and associated small lymph nodes (**Figure 1**). Further investigation was recommended. Single-balloon enteroscopy with cannula support was advanced 100 cm proximal to the ileocecal valve, where a circumferential neoplastic growth, approximately 10 cm in length, with an ulcerative appearance and a dirty purulent coating in the ileum was identified (**Figure 2**). Biopsy of the ileal lesion revealed a malignant lymphohematopoietic tumor with ulceration and necrosis; immunohistochemistry supported the diagnosis of an aggressive B-cell lymphoma. Immunohistochemical staining revealed the following results: Tumor cells were positive for CD20 (3+), while negative for CD3 (-, reactive T cells), CKpan (-), CK7 (-), CK20 (-), CDX-2 (-), CD56 (-), CgA (-), Syn (-). Other markers showed INI-1 (2+), BRG1 (3+), P53 (2+), and Ki-67 (approximately 80%+). EBER *in situ* hybridization (-). Then the patient completed positron emission tomography - computed tomography (PET-CT) showing primary small-bowel lymphoma, Lugano staging 1E. Considering the patient’s long-term anemia, we have decided to perform a partial ileal resection for the patient. During the operation, an 11-cm-long tumor was observed in the ileum, and it was adhered to the right posterior wall of the bladder and the sigmoid colon. After careful and slow dissection, we performed ileum tumor resection, sigmoid colon repair, and bladder repair (**Figure 3**). Postoperative pathology showed: “Partial ileum” resection specimen: Malignant tumor of the lymphohematopoietic system, with extensive necrosis. In view of the immunohistochemical findings, DLBCL is favored (germinal center subtype; ulcerative mass; size 11x7x1.5 cm); tumor tissue invades the entire intestinal wall to the serosal fibro-fatty tissue; no tumor cells are seen at both ends of the specimen and the circumferential margin, and no tumor tissue metastasis is seen in the peritoneal lymph nodes (0/8). Notes: 1. Immunohistochemical staining shows: Tumor cells are positive for CD20 (3+), CD79a (3+), Pax-5 (3+), Bcl-6 (2+), Bcl-2 5% (+), c-myc approximately 90% (+), CD10 (2+), CD3 (2+), CD43 (1+), CD30 (-), CKpan (-), GranB (-), TIA-1 (-), CD4 (-), CD8 (2+), Mum-1 (-), CD56 (-), ALK1 (-), Ki-67 approximately 80% (+). 2. *In situ* hybridization: EBER (-). 3. B-cell gene rearrangement: Detection of monoclonal band for IGH: D region (+); Detection of monoclonal band for IGK: A and B regions (+). 4. T-cell gene rearrangement: Detection of monoclonal band for TCRB (-); Detection of monoclonal band for TCRD (-); Detection of monoclonal band for TCRG (-) (**Figure 4**). The postoperative patient has made a good recovery. It is recommended that he be transferred to the oncology department for R-CHOP regimen. To date, five cycles have been completed. The treatment plan is to complete a total of six cycles, after which a formal response assessment will be performed.

### Literature search

Although previous reports on small intestinal DLBCL exist, cases involving MPC remain rare. We conducted a literature search using the terms “gastrointestinal diffuse large B-cell lymphoma” and “multiple primary cancers” or “simultaneous cancer” or “metachronous cancer” to retrieve relevant case reports in PubMed databases up to September 2025. We analyzed and summarized the clinical characteristics and outcomes of the three detected cases (13–15), with the summarized information presented in **Supplementary Table 1**.

## Discussion

Small bowel tumors (SBTs) are a rare entity, accounting for 0.6% of all newly diagnosed cancer cases in the United States, and only 3% of all gastrointestinal tumors, despite the small intestine being the longest part of the gastrointestinal tract (16, 17). SBTs have approximately 40 different histological subtypes, the most common of which are adenocarcinoma (30–45%), neuroendocrine neoplasms (20–40%), lymphoma (10–20%), and sarcoma (10–15%) (18, 19). Primary small bowel lymphoma most commonly involves the ileum. The main histopathological types include DLBCL, mantle cell lymphoma (MCL), follicular lymphoma (FL), marginal zone lymphoma (MALT), and Burkitt lymphoma (BL) (20, 21).

DLBCL exhibits diversity in both the underlying etiology of the disease and clinical treatment outcomes. Known risk factors for DLBCL include severe immune deficiency and organ transplant recipients. Factors leading to chronic immune deficiency include a variety of autoimmune diseases (such as Sjogren’s syndrome, systemic lupus erythematosus, rheumatoid arthritis, Crohn’s Disease), viral infections (such as human immunodeficiency virus, hepatitis C virus, hepatitis B virus, Epstein-Barr virus), and obesity. A family history of NHL/DLBCL, personal history of cancer, and multiple genetic susceptibility loci are also established risk factors for DLBCL (22–24). In this case, the onset of DLBCL in the patient may be related to a history of concurrent presence of lung cancer and cardia cancer. This is likely due to a variety of biological factors, including cancer predisposition syndromes and immune impairments.

Primary intestinal DLBCL (PI-DLBCL) is a malignant tumor that originates from lymphocytes in the intestine. The diagnostic criteria for PI-DLBCL are based on Dawson’s criteria (25). PI-DLBCL predominantly affected males, accounting for 68.18% of cases, with a median age at onset of 57 years (26). The clinical manifestations of PI-DLBCL typically include abdominal pain, ascites, hepatomegaly, splenomegaly, bleeding, general fatigue, anorexia, and weight loss. Some patients presented with acute surgical complications, such as perforation, bowel obstruction, ileal intussusception, or severe occult gastrointestinal bleeding (27–29). Another study has also shown that the most common symptoms in patients with DLBCL are occult blood in stool (70.0%) and weight loss (60.0%) (30). The main manifestations of the patient reported in this case are moderate anemia caused by occult blood in the stool and weight loss.

Most PI-DLBCL patients are not easy to diagnose clinically. The common diagnostic methods for PI-DLBCL include ultrasound, CT, PET-CT, magnetic resonance imaging (MRI) and endoscopy, such as esophagogastroduodenoscopy, colonoscopy and capsule endoscopy (28). A study has shown that in cases of primary small intestinal lymphoma, patients with DLBCL have longer involved bowel segments and more frequent bowel wall thickening on abdominal CT compared with those with indolent B-cell lymphoma (BCL) (p = 0.003). Moreover, compared with patients with indolent BCL, those with DLBCL were more likely to have aneurysmal dilation (p = 0.020) (30). The abdominal CT of the patient reported in this case was consistent with the above results, showing that the wall of the ileum was partially thickened, the surrounding space was blurred. At present, one of the most recommended examination methods for lymphoma patients is PET-CT, which has irreplaceable advantages. In cases of PI-DLBCL, PET-CT can show the accumulation of 18F-fluorodeoxyglucose metabolism at the site of the lesion, accurately locating the intra- and extra-intestinal lesions of PI-DLBCL. The PET-CT scan of this patient showed that the lymphoma was confined to the ileum and no involvement of other organs or surrounding lymph nodes was observed. In addition to emergency surgery, endoscopy is also an essential tool for diagnosing intestinal lymphoma. Endoscopic examination can visually observe the entire affected intestine, determine the exact location of the lesion, and perform biopsies to achieve an accurate preoperative diagnosis of PI-DLBCL.

A study has shown that approximately 15% of DLBCL patients have a history of MPC. Among the 123 DLBCL patients with MPC, 103, 16, and 4 had 1, 2, and 3 other primary malignant tumors respectively before receiving treatment for DLBCL. Gastric cancer was the most common, followed by colorectal cancer, lung cancer, prostate cancer, and breast cancer. Hematological malignancies were less common than solid cancers (31). This patient was found to have lung cancer and cardia cancer simultaneously before being diagnosed with DLBCL. The above study also showed that compared with patients without MPC, the overall survival (OS) and progression-free survival (PFS) of DLBCL patients with MPC are significantly shorter (P < 0.01 for both) (31).

The treatment for PI-DLBCL typically involves surgery, chemotherapy, and immunotherapy. The standard first-line chemotherapy regimen is R-CHOP (rituximab, cyclophosphamide, doxorubicin, vincristine, and prednisone) (32). However, in patients with NHL of the small intestine, chemotherapy can occasionally lead to perforation, which may be life-threatening. This increased risk is due to full-thickness damage caused by tumor or tissue necrosis following chemotherapy. A 37-year retrospective study of patients with gastrointestinal lymphoma found that 9% of patients experienced perforation, with 55% of perforations occurring after chemotherapy. The most common site of perforation was the small intestine (59%), followed by the large intestine (22%) and the stomach (16%) (33). Given the patient’s advanced age and the potential risks associated with chemotherapy, the initial step was to perform surgical resection of the diseased ileum. Complete resection of the affected area not only alleviates the patient’s symptoms but also reduces the risk of complications such as perforation, bleeding, obstruction, or intussusception. Moreover, early pathological diagnosis and clinical staging obtained from biopsies acquired through surgery are instructive for clinical diagnosis and treatment and do not increase the risk of patient mortality (34, 35). A study has shown that compared with surgery alone, in addition to surgically removing the primary site, systemic chemotherapy is associated with a significant increase in the survival rate of patients with non-metastatic primary small intestinal lymphoma. Therefore, the recommended treatment for PI-DLBCL is a combination of surgery and adjuvant therapy (including radiotherapy, chemotherapy, and targeted therapy), which can lead to better prognosis (36). After this patient’s surgery, we recommended that he visit the oncology department for chemoimmunotherapy.

The principal novelty of this case is twofold. First, to our knowledge, this represents a rare documented instance of a patient with synchronous dual solid tumors (cardia and lung adenocarcinoma) subsequently developing a primary small intestinal DLBCL presenting with unexplained anemia as the initial symptom. This sequence underscores the diverse spectrum of MPC and reinforces that cancer survivors require lifelong vigilance for new primary malignancies across all lineages, including hematologic ones. Second, and more importantly, this case serves as a compelling teaching tool regarding diagnostic reasoning and cognitive bias in oncology. The patient’s complex history created a high risk for “anchoring bias,” where clinicians might prematurely attribute his anemia to his past cancers or their treatment effects. The absence of typical DLBCL symptoms (e.g., pain, bleeding) further allowed this bias to persist. Our diagnostic odyssey - from unremarkable standard endoscopies to the pivotal findings on capsule endoscopy and enteroscopy - illustrates the necessity of persisting with a broad differential diagnosis and utilizing advanced diagnostic modalities when initial tests are inconclusive. We propose that such cases necessitate a deliberate, two-pronged diagnostic mindset: actively investigating for recurrence or complications of known diseases while simultaneously screening for new, unrelated primaries.

### Clinical implications

Based on our experience, we propose the following key considerations for clinicians managing similar cases. First, in cancer survivors presenting with unexplained, non-specific symptoms such as anemia, a systematic workup must actively include the possibility of a new, metachronous malignancy (both solid and hematologic) alongside evaluation for recurrence or treatment sequelae. This deliberate mindset is the primary defense against anchoring bias. Second, when standard endoscopic evaluations (esophagogastroduodenoscopy and colonoscopy) are unrevealing in the context of persistent anemia, capsule endoscopy should be promptly considered as a non-invasive tool to screen the entire small bowel. Following a tissue diagnosis, PET-CT is indispensable for accurate staging of lymphomas, defining disease extent, and guiding treatment planning. Third, for localized primary small intestinal DLBCL, surgical resection prior to chemotherapy can serve a dual purpose. It provides definitive histopathological diagnosis, reduces tumor burden, and crucially, mitigates the risk of perforation or bleeding that can occur during chemotherapy-induced tumor lysis.

### Limitations

While bone marrow biopsy is an important tool in the staging of lymphoma. However, in this case, the patient and family declined the procedure after considering the following factors: (1) the whole-body PET-CT scan showed no evidence of bone marrow involvement; and (2) peripheral blood tests revealed only anemia, without other cytopenias or abnormal cells indicative of bone marrow infiltration. We acknowledge that the lack of biopsy confirmation is a limitation in definitively establishing Lugano staging IE. Nevertheless, based on this comprehensive non-invasive assessment, the clinical team believes the likelihood of limited-stage disease is high.

## Conclusion

This case highlights that in patients with prior malignancies, non-specific symptoms like anemia should trigger a systematic workup that explicitly includes the possibility of a metachronous hematologic cancer. Although the exact pathogenesis is not fully understood, potential contributing factors may include genetic susceptibility, immune microenvironmental dysregulation, and shared carcinogenic exposures. Unexplained anemia should prompt thorough investigation, including endoscopy, radiology, pathology, and immunohistochemical characterization (CD20, CD79a, Pax-5, Ki-67, etc.) for accurate differentiation. Prompt diagnosis and appropriate treatment, as demonstrated in this case, are crucial for managing such complex presentations and improving patient outcomes.

## Data Availability

The original contributions presented in the study are included in the article/**Supplementary Material**. Further inquiries can be directed to the corresponding authors.
