# Supplementary material for: Unmasking small-bowel DLBCL in an elderly patient with simultaneous multiple primary cancers through anemia: a case report and literature review
Source: Front Oncol. 2026 Feb 19;16:1706363. doi: 10.3389/fonc.2026.1706363 (PMC12960075; doi:10.3389/fonc.2026.1706363)
Supplement: Supplementary file 1 [file DataSheet1.docx]

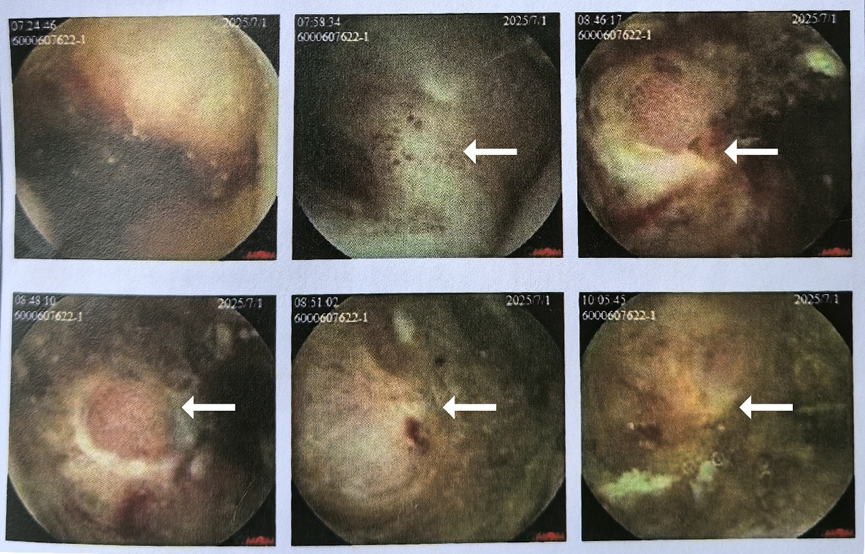


eFigure 1 Capsule endoscopy performed at the local hospital revealing that marked mucosal edema, extensive erosions and deep ulcers was seen in the ileum (white arrows)

eTable1 A summary of three cases of gastrointestinal DLBCL combining MPC reported in the literature from 1995 to 2025

| Year | Age | Sex | MPC | Synchronous/ metachronous | Location of DLBCL | Complaint | Clinical treatment | Follow-  up |
| --- | --- | --- | --- | --- | --- | --- | --- | --- |
| 2019 ^[13]^ | M | 78 | sigmoid colon adenocarcinoma | synchronous | ascending colon | palpable lumps in the abdomen | laparoscopic RH +sigmoid colon resection | 1 month |
| 2021^[14]^ | M | 52 | oral squamous cell carcinoma | metachronous | terminal ileum | AP+bloody stools | ileum resection +R-CHOP | 4 years |
| 2025^[15]^ | M | 82 | rectum adenocarcinoma | synchronous | ascending colon | irregular bowel movement+AP | laparoscopic anterior resection of the rectum +open RH | 2 months |

M: Male; AP: Abdominal pain; RH: right hemicolectomy; R-CHOP: rituximab, cyclophosphamide, doxorubicin, vincristine, and prednisone
